# Three-Dimensional Smooth Particle Hydrodynamics Modeling and Experimental Analysis of the Ballistic Performance of Steel-Based FML Targets

**DOI:** 10.3390/ma15103711

**Published:** 2022-05-22

**Authors:** Andrzej Kubit, Tomasz Trzepieciński, Radosław Kiciński, Kamil Jurczak

**Affiliations:** 1 Department of Manufacturing and Production Engineering, Rzeszow University of Technology, al. Powst. Warszawy 8, 35-959 Rzeszow, Poland; tomtrz@prz.edu.pl; 2 Mechanical and Electrical Engineering Department, Polish Naval Academy, 81-103 Gdynia, Poland; r.kicinski@amw.gdynia.pl (R.K.); k.jurczak@amw.gdynia.pl (K.J.)

**Keywords:** ballistic impact, ballistic shield, FML, smooth particle hydrodynamics, FEM-SPH

## Abstract

In this paper, shields made of 1.3964 stainless steel bonded to a fiber laminate were subjected to ballistic impact response of 7.62 × 51 mm ŁPS (light projectile with a lead core) projectiles. Additionally, between the steel sheet metal and the laminate, a liquid-filled bag was placed, which was a mixture of ethylene glycol (C_2_H_6_O_2_) with 5 wt.% SiO_2_ nanopowder. Numerical modeling of the projectile penetrating the samples was carried out using the finite element method in the Abaqus program. The elasto-plastic behavior of the projectile material and the component layers of the shields was taken into account. Projectile penetration through glycol-filled bag has been performed using the smooth particle hydrodynamics technique. The morphology of the penetration channel was also analyzed using a scanning electron microscope. For the shield variant with a glycol-filled bag between the steel and laminate plates, the inlet speed of projectile was 834 m/s on average, and 366 m/s behind the sample. For the variant where there was no glycol-filled bag between the steel and laminate plates, the inlet and outlet average velocities were 836 m/s, after 481 m/s, respectively. Referring to the steel-glycol-laminate and steel-laminate variants, it can be concluded that the laminate-glycol-laminate is more effective.

## 1. Introduction

Due to their high specific strength and high strength to weight ratio compared to traditional materials, composite materials are more and more often used in transport, construction [1] and aviation industry [2]. Fiber metal laminates (FMLs) are multifunctional hybrid materials that exhibit excellent impact resistance and improved energy absorption properties compared to a structure made of metal or composite alone [3]. The brittleness of composites and the associated tendency to fracture make the resulting structures very sensitive and susceptible to sudden localized dynamic loads, such as ballistic impacts and aviation bird strikes.

Lightweight multilayer ballistic shields are increasingly used to increase the blast resistance of light ships, cars, airplanes and bulletproof jackets [4,5]. For many years, high-strength steel plates have been used as shields. Currently, with the development of ballistic shields consisting of, among others, composites, low-density multi-layer shields seem more and more promising. Lightweight ballistic shields are a system of several or even a dozen or so layers of different materials, combined or separated, forming the so-called layered composite structure [6]. D’entremont et al. [7] emphasized the role of the adhesive bond in forming the protective properties of a material. Adhesives based on epoxy resins or cyanoacrylate adhesives are most often used to join individual armor layers [6].

The suitability of the materials used for multilayer structures to absorb the energy of the projectile is carried out by means of impact and ballistic tests. The basic tests include the measurement of the resistance force of the shield against projectile, the test of the projectile’s velocity in front of and behind the sample (energy measurement), and the measurement of the pendulum swing angle at the moment of penetration of the test sample by the projectile. Composites have high ballistic resistance and show different mechanical properties at different deformation rates [8].

In order to improve the ballistic resistance of composite materials, many improvements have been developed, such as the development of high-strength, ultra high molecular weight polyethylene (UHMWPE) fibers [9], the development of ceramic-based laminates as armor covers [10], and the overlapping of layers of brittle and ductile material in an appropriate arrangement stack [3]. FMLs consist of alternating composite plies and metallic sheets and due to superior fatigue behavior and impact resistance and damage tolerance they are excellent candidates for hulls of marine transportation systems. Blast loaded FML panels are able to distribute the loading more evenly across the panel than monolithic metal configurations [11]. Moreover, over the past few years, composite/aluminum hybrids such as FMLs have begun attracting interest for use in blast-attenuating structures [12]. The relatively high cost of aluminum alloys caused interest in the development of steel-based FMLs [11].

The ballistic properties of FMLs depend on many factors, such as the shape and incidence angle of the projectile, the impact energy and the stacking order [13]. The impact behavior of composite materials, including FMLs, is mainly attributed to impact velocity. According to the velocity, the impact event can be divided into low-velocity (≤11 m/s), high-velocity (≥11 m/s), ballistic (≥500 m/s), and hypervelocity impact (≥2000 m/s) [14].

In the collision at high speed, transverse shear stresses regulated by transverse shear waves dominates. On the other hand, during the collision with low velocity, the stresses in the plane, regulated by bending waves, dominate [3]. Studies on the effect of fiber thickness and orientation on the energy absorption of laminates and ballistic limit under high impact velocity conditions were carried out by Sirkawar et al. [15]. They found that the 0/90° laminate showed the best ballistic resistance and strain change at break in different directions of the fibers. The ballistic limit and the energy absorption of glass fiber reinforced polymers (GFRPs) significantly depend on the shape of the penetrator [16].

Various parameters such as the fibre material (glass, carbon, aramid and polypropylene), reinforcement types (woven or unidirectional), the number of metallic layers, and the material of metallic layers (aluminium alloys, steels and titanium) have been analysed by Abdullah and Cantwell [17,18], Ahmadi et al. [19], Chen et al. [16], Li et al. [20], Sharma and Khan [21] using rigid projectiles shot via gas gun setups. The permanent deformations, external and internal damage patterns, and different failure modes have been investigated by the mentioned researchers. The interaction among failure area, ballistic limit velocity, damage modes, and various mechanisms of energy absorption based on projectile’s rigidity or deformability have been investigated by Sangsefidi et al. [22]. Three sets of FML samples containing 4, 8, and 16 composite plies and two facing 2024-T3 aluminium alloy plates have been considered. It was found that by increasing the deformability of the projectiles, the amount of absorbed energy for perforating the targets and the ballistic limit velocity of the FML targets increase. Abdullah and Cantwell [23] studied the high-velocity impact response of thermoplastic–matrix FMLs. The impact resistances of the various laminates based on the 2024-T3 and 2024-O were compared by determining their specific perforation energies. The ballistic response of FMLs based on the high-strength 2024-T3 alloy out-performed their 2024-O counterparts. Moreover, it was found that the self-reinforced polypropylene FMLs offer a slightly lower perforation resistance than FMLs based on the glass reinforced polypropylene composite. Kikakis, et al. [24] investigated the influence of the mechanical properties of different aluminium alloys (2024-O, 2024-T351, 6061-T6, 7075-T6, and 7039) on the ballistic resistance of glass-reinforced FMLs. It was concluded that the ballistic limits of the FML panels can be substantially affected by the constituent aluminium alloy. Ramadhan et al. [25] investigated the high velocity (400 m/s) impact response of composite laminated plates has been experimentally investigated using a nitrogen gas gun and a cylindrical shape of 7.62 mm diameter steel projectile. FML structures based on Kevlar-29 fibre/epoxy-alumina resin with different stacking sequences of 6061-T6 aluminium alloy plates were considered as targets. It was fund that the increasing of the amount of the energy absorption obtained was 7.3% and 2.7% of the back to middle and back to front 6061-T6 aluminium alloy sheet stacking sequence, respectively. Investigations of blast performance of the glass/polypropylene FML samples shown the presence of delamination and localised fibre fracture in the composite plies, extensive matrix microcracking and thinning in the aluminium layers [26]. The adhesion at the steel-composite interface in the FMLs was investigated by Langdon and Rowe [11] using three point bending and it was shown that a single heating/pressing operation had better performance than the two-stage methods and those involving a third party adhesive. Inelastic deformation and debonding failure of the steel-composite interfaces were similar to those exhibited by blast loaded FMLs manufactured with the same composite material but with aluminium alloy sheets. Balkumar et al. [27] show that the thickness of the aluminium-based FML plate and impactor geometry were the significant process parameters related to the response of low velocity impact analysis of target plate. A comprehensive review on impact properties of FMLs is provided by Sadighi et al. [28].

Particularly helpful in the analysis of FMLs responses to dynamic impact is numerical modeling based on theorems of continuous media mechanics, fracture mechanics and computer-based methods of solving equations of motion [29]. In the literature many examples of the use of numerical modeling in the study of the behavior of multilayer structures can be found. These methods include the finite element method, finite element method coupled to smooth particle hydrodynamics (FEM-SPH), elastic bond-based peridynamics (PD), generalized particle algorithm (GPA) and discrete element method (DEM). FEM is unsuitable for the description of discontinuity of cracks initiation and propagation [30]. The disadvantages of the FEM may be compensated for by the use of mesh-free methods. The FEM-SPH method has proven to be very effective for brittle fracture simulation, especially for high-speed impact conditions [31]. In particular they can easily handle large deformations, since they do not need predefined connections between nodes. Guan et al. [32] investigated the impact response of FMLs based on a polypropylene fiber/polypropylene matrix composite and two types of aluminum alloy (2024-O and Al 2024-T3) using explicit finite element model. Shear and tensile failure criteria were employed to model failure in the aluminum plies by specifying failure tension cut-off stresses. To model non-perforation failure of FMLs subjected to projectile impact, the combination of tension cut-off stresses and shear strain failure may be appropriate. The ballistic resistance of GLARE 4A FMLs subjected to high velocity impact has been investigated by Bikakis et al. [33]. They considered simultaneous existence of various impact damage mechanisms of laminate plies. It was numerically found that the ballistic limit of the GLARE 4A panels becomes higher as their thickness increases. Yaghoubi and Liaw [34] build finite element model of ballistic perforation of GLARE 5 (3/2) FMLs. It was found that cross-ply composites dissipate more energy than unidirectional composites. Perforation failure of various stacking configurations of layers of woven glass fiber in a polypropylene matrix and aluminum alloy sheets subjected to the localized high intensity blast loading was studied by Sitnikova et al. [35]. The results indicate that the failure criteria and material constitutive models are able to represent a number of high strain-rate failure features in the FMLs, such as perforation failure, multiple debonding and petaling. Numerical analyses conducted by Karagiozova et al. [36] highlighted the importance of accurate finite element modelling of the applied pressure, since the initial deformation phase is highly sensitive to spatial distribution of applied pressure.

Predicting the behavior of a multilayer shield on the basis of the results of numerical analyzes requires the calibration of the numerical model based on the results of experiments. Zhang et al. [37] established a nonlinear dynamic Finite Element Method (FEM) model to analyze energy absorption and FML damage due to oblique impacts. They found that the impactor’s residual velocity and the energy absorption by FMLs were significant for the initial impact velocity and impact angle. Ansari et al. [38,39] developed three-dimensional finite element-based model for progressive failure analysis of GFRP laminates and showed failure evolution and laminate propagation due to projectile impact. Damage to the GFRP laminate at various angles of incidence, boundary conditions and shapes of the nose of the projectile is also discussed.

Chen et al. [40] investigated ballistic resistance of GFRP laminates subjected to an impact of 150 m/s. A three-dimensional model combining the strain rate effect and the Hashin failure criterion was established. It was found that the stacking sequence contributes a stronger influence on the maximum deflection of the GFRP laminates. Vo et al. [41] numerically investigated influence of different aluminum alloys in FMLs and found that FML with higher yield modulus and strength (7075-T6) provide better explosion resistance among other aluminum alloys (2024-O, 2024-T3, 6061-T6) tested. It was also found that the internal debonding is dependent on the yield strength of the aluminum alloy. Chai et al. [3] applied FEM in order to investigate the dynamic perforation behavior caused by the ballistic impact loading of titanium-based fiber metal laminates (TFMLs) made from different stacks. The addition of an additional titanium layer and a CFRP layer to create TFML-3/2 compared to TFML-2/1 increased the weight of the target by 58%. The thicker thickness of TFML-3/2 increased ballistic speed by 32% (in the experiment) and 37% (in the numerical model) compared to the TFML-2/1. Sitnikova et al. [35] have reported that most of the failure modes of FMLs for loading with high-intensity blast impact have been captured by their finite element model. The penetration resistance of elastomer matrix Kevlar composites during impact of spherical projectile was investigated numerically by Asemani et al. [42]. They found that elastomeric composites can cause to reduce the damage area and increase energy absorption. Yang et al. [14] developed FEM model to simulate the effect of thickness and fiber stacking sequence on the ballistic response of FMLs at speeds 500 m/s < v ≤ 900 m/s. It has been concluded that the fiber stacking sequence in FMLs has very limited influence on the impact performance.

One of the methods to improve ballistic response of variable-stiffness armors in the field of military protection are magnetorheological fluids (MRFs) [43]. The other low-cost method of improvement of the ballistic response of target is to use high-density suspensions. MRFs are suspensions of magnetic particles dispersed in a non-magnetic carrier liquid. The concept of liquid-filled bags in smart protective armors arises from their characteristic ability to dissipate and absorb energy by varying the magnetic field intensity [44]. In order to improve the absorption of energy by composite panels, various modifications have been made in the geometry of its core [45] and application of the shear thickening fluid-filled core of a composite panels [46]. Shear thickening *fluid* (STF) is a kind of intelligent ballistic-resistant material that shows non-Newtonian behavior [47]. When subjected to a high-speed impact of projectile, the viscosity increases rapidly and gradually returns to a colloidal state after the external force disappears [47,48]. Chatterjee et al. [49] investigated the steady-state rheological behavior of the formulated STF using spherical silica nanoparticles dispersed in polyethylene glycol of molecular weight 200 g/mol (PEG-200). Panels with polyethylene glycol-filled core could absorb an energy of 81.3% whereas the hollow sandwich panel could absorb only 56.3% of the impacted energy. According to the Abtew et al. [5], the ballistic performance of composite panels could also be improved by impregnated the fabrics with a colloidal STF (silica particles (450 nm) dispersed in ethylene glycol). The solid particles dispersed in STFs may be synthetic (polymers) or naturally occurring mineral, including calcium carbonate, SiO_2_ or other oxides [50]. Classification of dispersed phases and dispersion fluids in STFs investigated in the last decade were provided by Zhang et al. [47].

In this paper, the samples made of 1.3964 stainless steel bonded to the fibre laminate were subjected to ballistic impact response of 7.22 mm ŁPS projectiles. Additionally, between the steel sheet metal and the laminate, a liquid-filled bag was placed, which was a mixture of ethylene glycol (C_2_H_6_O_2_) with 5 wt.% SiO_2_ nanopowder. Numerical modeling of the projectile penetrating the samples was carried out using combined the finite element method and smooth particle hydrodynamics technique. The elasto-plastic behavior of the projectile material and the component layers of the shields was taken into account. The changes in velocity of the projectile during its passage through different variants of FML-based shields were compared.

## 2. Materials and Methods

### 2.1. Materials

The target was three variants of samples with dimensions of 100 × 100 mm, which were shot through a projectile, caliber 7.62 × 51 mm (Mesco S.A., Skarżysko-Kamienna, Poland). Projectile consists of a lead core and a copper jacket. The dimensions of the samples corresponded to the dimensions of the sample mounting head in a B571 Optical Target System used in ballistic testing by manufacturer of the service ammunition (Zakłady Mechaniczne in Tarnów, Poland).

**Figure 1:**
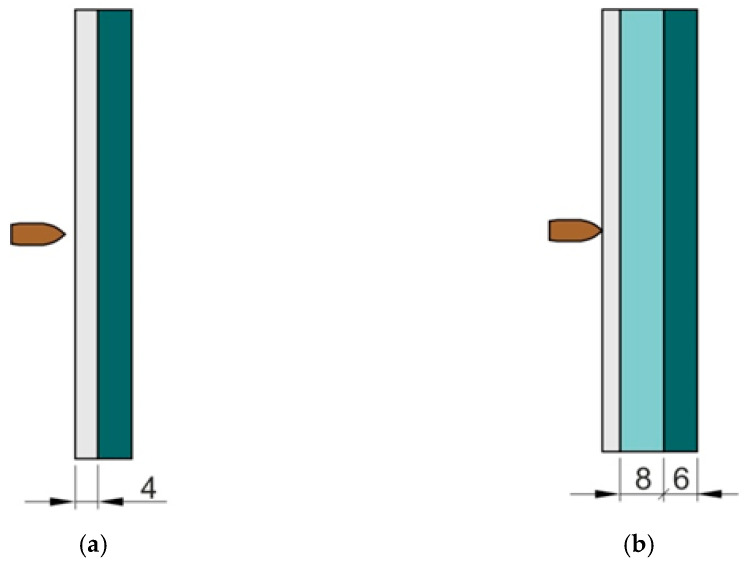
Variants of the samples tested in experimental tests (units in mm): (**a**) steel-laminate and (**b**) steel-glycol-laminate.

In the first variant the 1.3964 stainless steel plate with thickness of 4 mm and a laminate plate with thickness of 6 mm (Figure 1a) were joined together using Araldite 2014-2 (Huntsman Advanced Materials, Everberg, Belgium) epoxy based adhesive (the manufacturer recommended resin and hardener mixing ratio 100:50 by weight was used). The 1.3964 steel is a non-magnetic stainless steel with austenitic structure, characterized by high mechanical properties and very good corrosion resistance. The grade shows comparable corrosion resistance to other austenitic steels, high yield strength, and stability of mechanical properties at low temperatures. This steel is also resistant to inter-crystalline corrosion, sea water, salts, chlorides, sulfur compounds, nitric acid, formic acid and phosphoric acid [51]. The physical and mechanical properties of 1.3964 sheet metal allow it to be used as ballistic shields used in the marine environment. The Brinell hardness test was performed, the hardness was measured at three points on the surface of the sheet, the average value was 252 HB.

Table 1 shows the chemical composition of this material according to the metallurgical certificate provided by the supplier.

The laminate plate was made of 20 layers of aramide fabric with a grammage of 230 g/m^2^. The fabric layers were joined according to the orientation [0/90/45/0/90/-45/0/90/45/0/90/-45/0/90/45/0/90/-45/0/90]. Epoxy resin MGS 285 with a dedicated hardener was used as the matrix using resin and hardener mixing ratio equal 100:40 by weight. The laminates were post-cured at an elevated temperature 140 °C for 90 min. In the second analyzed variant of the sample, a liquid-filled bag with thickness of 8 mm (Figure 1b) containing a mixture of ethylene glycol with 5 wt.% SiO_2_ nanopowder was placed between the steel and laminate plates.

### 2.2. Experimental Setup

**Figure 2:**
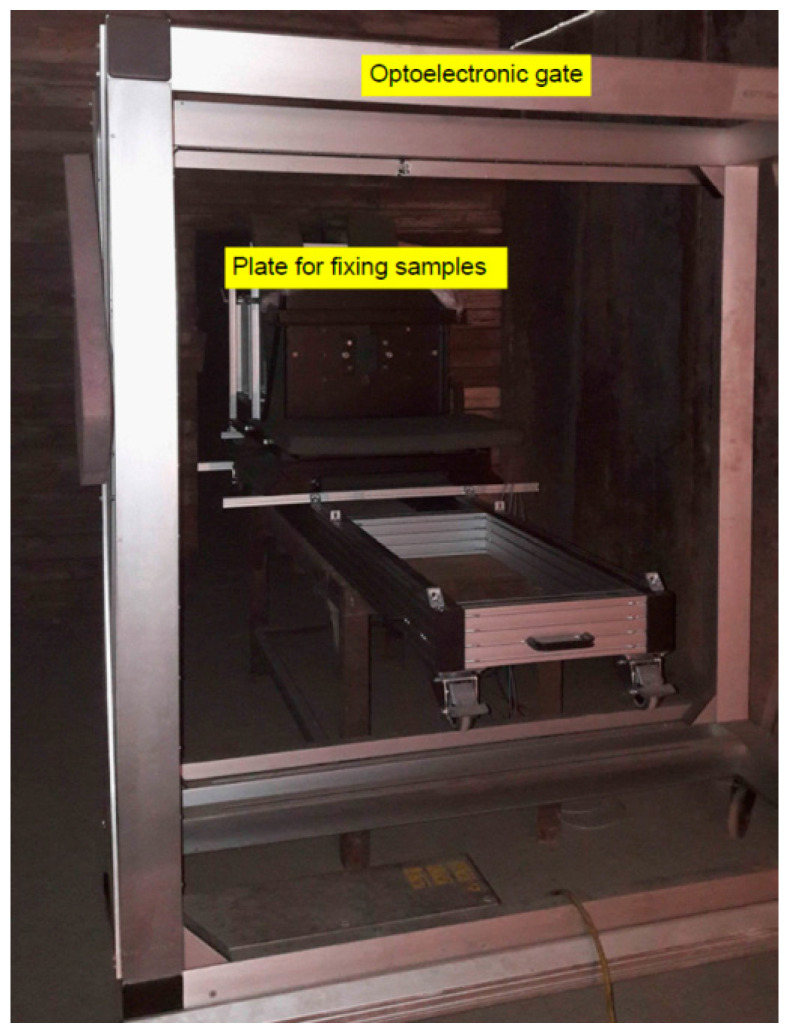
View of the optoelectronic gate to measure the velocity of projectiles before penetrating the sample.

**Figure 3:**
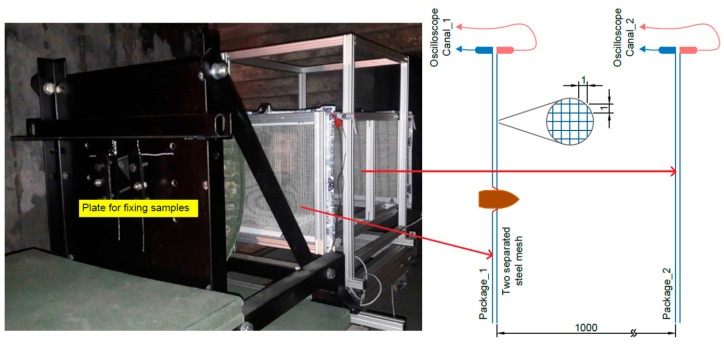
Stand for shooting samples.

**Figure 4:**
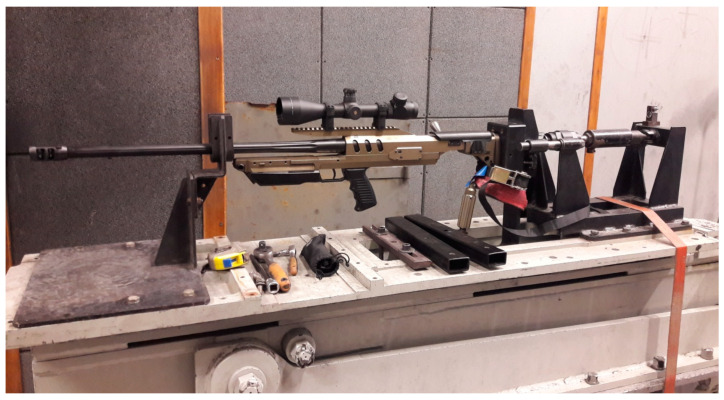
Launcher with mounted ALEX .308 riffle.

The tests of perforation resistance of the samples were carried out on the basis of internal materials certification procedures for the defense industry in Zakłady Mechaniczne in Tarnów. Projectile velocity before hitting the sample was measured with a B571 Optical Target System (Figure 2). On the other hand, the measurement of the impactor’s residual velocity after shooting the sample was made with the use of mesh detectors, the principle of which is based on the fact that at a distance of 1 m from each other, packages of two separated steel mesh constituting an open electrical system were placed. At the moment of shooting through such packages, the originally separated meshes closing the electric circuit. Such a phenomenon was registered with the use of a digital oscilloscope, in this way, the moments of short circuit were recorded. Based on the time interval of both short circuit and the known distance between the mesh packets, the projectile velocity was determined. The first package of steel mesh was placed 500 mm behind the target. The principle of operation of such a measurement is schematically shown in Figure 3. The samples were mounted symmetrically in the central part of the base frame. The ALEX .308 ZMT HS rifle, which uses the .308 Winchester ammunition, which is a variant of the BOR-7.62 military sniper rifle, is mounted in the technological holder of the launcher (Figure 4), 20 m away from the target.

### 2.3. Numerical Modeling

#### 2.3.1. Constitutive Equation

On the basis of the conducted experiments, a projectile penetration simulation of the aluminum alloy-based FMLs was carried out with the use of FEM. In this case, the constitutive equation of the solution is complicated and can take the following form [52]:

(1) $$M\left(U,n\right)\ddot{U}+C\;\dot{U}+K\left(U,\;{\dot{\varepsilon }}_{\mathrm{pl}},\varepsilon_{\mathrm{failure}},n\right)U=F\left(m_{\mathrm{pr}},\alpha ,v,t,\;{\dot{\varepsilon }}_{\mathrm{pl}},\varepsilon_{\mathrm{failure}},C_{\mathrm{int}},\mu \ldots \right)$$

where: K is stiffness matrix; M is inertia matrix; C = αM + βK is damping matrix (α, β—constants); U, $\dot{U}$, $\;\ddot{U}$ are the vectors of displacement, velocity and acceleration, respectively; F is vector of loads; ${\;\dot{\varepsilon }}_{\mathrm{pl}}$ is vector of strain rate; ε_failure_ is vector of failure deformation; n is number of material layers; v is projectile speed before impact; t is time; α is inclination angle, m_pr_ is projectile mass; C_int_ are interactions and contact forces between colliding FEM elements; and μ is the coefficient of friction.

Equation (1) shows the multitude of factors influencing the solution of problems related to dynamic loads. A constructor facing a problem in this area must find a compromise between the accuracy of the solution and the number of factors taken into account. Due to the very short loading time, great attention should be paid to the selection of the size of the finite elements and the time step. Calculations in the dynamic range are complex and long-term, therefore for large structures, with an incorrectly selected time step, the nature of the load depending on the strain rate may significantly distort the results.

#### 2.3.2. Model Geometry

**Figure 5:**
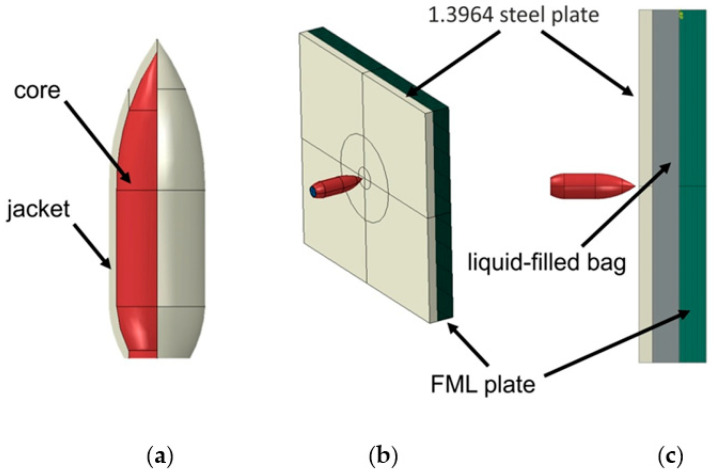
Geometry of the problem: (**a**) projectile, (**b**) model without a fluid-filled bag, (**c**) model consisted a fluid-filled bag between FML and steel plates.

Target in the numerical model consisted of a 100 × 100 mm plate which is hit by a deformable projectile (Figure 5a). Three variants of geometry of the target were considered. In the first one 1.3964 steel and a laminate were joined together (Figure 5b). Inside the laminate 20 layers were modeled. In the second model fluid-filled bag was placed between the steel plate and laminate (Figure 5c). The variant where fluid-filled bag between two laminate plates was also considered. Projectile consists of a lead core and a copper jacket.

**Figure 6:**
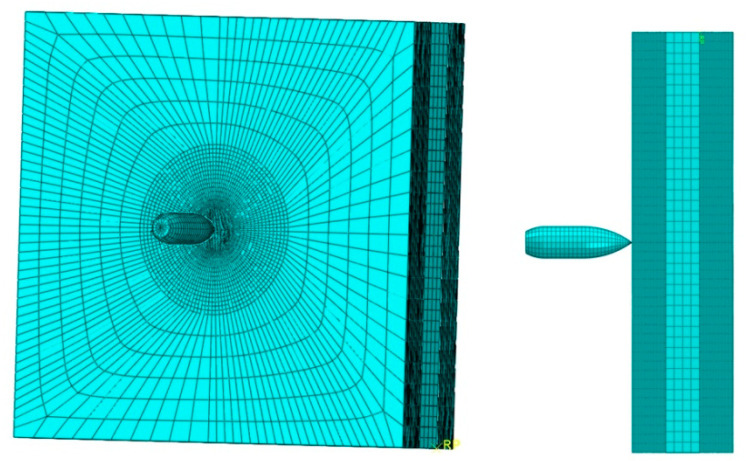
FEM mesh for the most complex variant (laminate-glycol-laminate).

In the considered variants of geometry, eight-node solid elements with different mesh sizes were used for modeling. The mesh was densified at the point of intended contact of plate with the projectile. The sample model was prepared and discretized in such a way that the finite element size at the point of contact with the projectile was L = 0.0005 m. In the most complex case, the task consisted of 235,604 finite elements, as shown in Figure 6.

#### 2.3.3. Boundary Conditions

**Figure 7:**
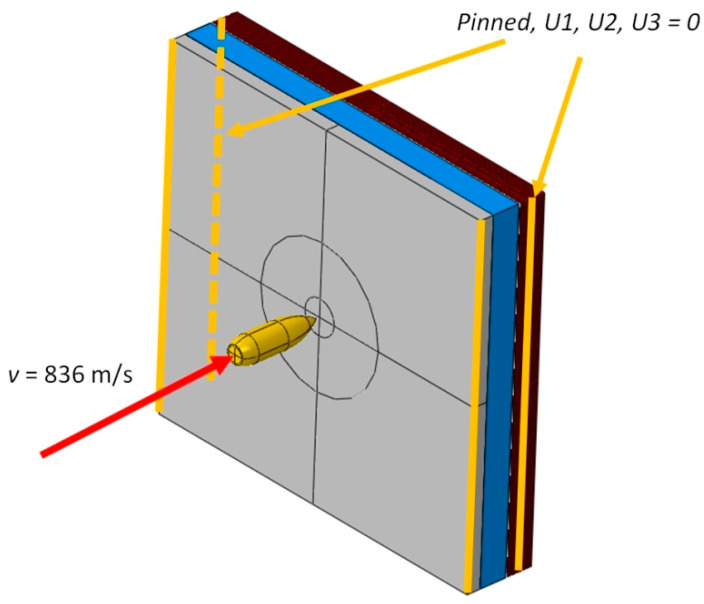
Boundary conditions for variant laminate-glycol-laminate.

After modeling the geometry of the task, the boundary conditions were assigned. According to the measurements from the experiment, the speed of the projectile at the moment of hit in sample was assigned 836 m/s. Then the global finite element contacts were defined. Due to the fact that new contact surfaces appear during the destruction of individual elements, interactions should be defined for each finite element. The friction coefficient of μ = 0.55 was assumed between the elements. The two edges of the steel and FML plates were prevented from moving in each direction. The boundary conditions of the modeled problem are presented in Figure 7.

#### 2.3.4. Material Properties

The next stage of creating a finite element model is assigning material data to individual elements. The material data were obtained on the basis of own experiments and literature. In most cases, a Johnson-Cook (J-C) material model describing both the plastic properties and the destruction conditions of a given material is used. In the case of the plastic material model, the J-C equation is presented as [53,54,55,56,57]:

(2) $$\sigma =\left(A+{B\varepsilon }^{n}\right)\left(1+\mathrm{Cln}\left(\frac{\dot{\varepsilon }}{{\dot{\varepsilon }}_{0}}\right)\right){\left(1-\frac{T-T_{0}}{T_{m}-T_{0}}\right)}^{m}$$

where σ is the equivalent stress, A is the field stress of the material under reference conditions, B is the strain hardening constant, ε is equivalent plastic strain, n is strain hardening exponent, C is the strengthening coefficient of strain rate, $\dot{\varepsilon }$ is deformation strain rate, ${\dot{\varepsilon }}_{0}$ is the reference strain rate, T is deformation temperature, T_0_ is ambient temperature, Tm is melting temperature, and m is the thermal softening coefficient.

The presented Johnson-Cook material model is a description of the material behaviour as a function of strain rate and temperature. It is suitable for the description of phenomena in which the strain rate does not exceed 10^5^ s^−1^ [58]. The J-C failure criterion is a special variety of the failure prediction model based on the nucleation and enlargement of voids in plastic metals. It is a function of the triaxial stress and strain rate as well as temperature [59,60]:

(3) $${\bar{\varepsilon }}_{D}^{\mathrm{pl}}\left(\eta ,{\bar{\dot{\varepsilon }}}^{\mathrm{pl}},\;\hat{T}\right)$$

The strain for which the J-C criterion is met is given by the Equation (4) [61]:

(4) $${\bar{\varepsilon }}_{D}^{\mathrm{pl}}=\left[d_{1}+d_{2}\exp\left(-d_{3}\eta \right)\right]\left[1+d_{4}\ln\left(\frac{{\bar{\dot{\varepsilon }}}^{\mathrm{pl}}}{{\dot{\varepsilon }}_{0}}\right)\right]\left(1+d_{5}\hat{T}\right)$$

where *d*_1_, *d*_2_, and *d*_3_ are material constants describing the quasi-static failure of a material; *d*_4_ is material constant depending on the strain rate; *d*_5_ is the temperature-dependent material constant; ${\dot{\varepsilon }}_{0}\;$ is the speed of quasi-static deformation ${\dot{\varepsilon }}_{0}=0.0001{\;s}^{-1}$; and $\;\hat{T}$ is the equivalent temperature coefficient defined as:

(5) $$\;\hat{T}=\{\begin{array}{c} 0\\ \frac{T-T_{0}}{T_{\mathrm{top}}-T_{0}}\\ 1 \end{array}\;\begin{array}{c} \mathrm{for}\;T<T_{0}\\ {\mathrm{for}\;T}_{0}\leq T\leq T_{m}\\ \mathrm{for}\;T>T_{m} \end{array}$$

The use of advanced failure criteria is also justified due to the mechanism of the formation of adiabatic shear bands. The interaction of the tested material with the projectile is complicated due to the high interaction energy and strain rate. This interaction causes fracture of the material and can also induce significant changes in the material in the area of interaction. A characteristic feature of dynamic deformation in metallic materials, which occurs at a deformation rate higher than 10^3^ s^−1^, is the formation of areas with extremely high accumulation of deformation energy, called adiabatic shear bands. In these areas, the dynamic strain energy generated in the shear bands is almost entirely used for phase and structural transformations [62].

To complete the fully nonlinear analysis, a model of failure and deformation for the projectile is also considered. Most studies in this area propose to use the projectile as a non-deformable body in order to reduce the computation time. The validity of such a solution was verified in [63]. A series of simulations were carried out with the use of a projectile modeled as an elastic, elastic-plastic and elastic-plastic body with the failure criterion. The results of the simulations show that the fully deformable projectile model with failure criterion which has been taken into account as described is the most accurate. Material data for the projectile (Table 2) were obtained from the literature [64].

Based on the tests included in [63,65], the parameters in the material model of 1.3964 steel (Table 3) were determined with the use of the J-C visco-plasticity and failure equations. The material data for the laminate was adopted on the basis of literature [66] and the material issue list. Laminate is an orthotropic material, therefore it is characterized by material constants depending on the direction of the load force. Material parameters of laminate are presented in Table 4.

Failure of laminates is complex and difficult to model in the case of solid finite elements. Therefore, it was decided to use a simplification in which failure was assigned for the triaxiality factor equal to η = ± 0.33. This simplification does not always work in practice, but in the considered case it is sufficient to achieve simulation results similar to the experiment. Failure parameters for the laminate were modeled as ductile damage and they are as follows:

1. Failure strain ε_failure_ = 0.03;
2. Failure displacement u_failure_ = 0.0003;
3. Friaxiality factor η = ± 0.33.

The above parameters were determined using an experiment in which steel samples joined to the laminate were shoot through. These data can be refined, but require more laminate testing. The combination of two completely different media (fluid—solid body) in numerical quantification is a complex issue and requires careful selection of many factors. One way of modeling fluids are meshless methods. One of the most popular methods is the smooth particle hydrodynamics (FEM-SPH) method, which is used to solve boundary problems. It uses a specific type of discretization, in which neighboring nodes remain adjacent to each other during the calculations [67,68]. The FEM-SPH method is effective when applied to fluid–structure interaction (FSI) simulations in the case of complex and time-varying contact zones [69,70], which makes it effective for simulating the shoot through process with plastic deformation and the failure criterion.

The FEM-SPH method was derived for problems described by partial differential equations in space of variables, such as density, energy, and velocity. An analytical solution to such a system of equations is usually impossible. The numerical solution requires first discretization of the domain on which the equations were defined, and then approximation for each point of each variable from the permissible function space and its derivatives. This method is based on the interpolation theory. Unlike FEM, in which the values of individual model variables are calculated using the shape function, the FEM-SPH method discretizes the continuous distributions of parameters such as liquid density or pressure by replacing them using estimates at the assumed interpolation kernel. In this paper, the Hugoniot and Rayleigh state equation (called the shock Hugoniot in the u_s_-u_p_ plane) was used to describe the behavior of the fluid during an impact with a projectile (transfer of a shock wave) in the form [71]:

(6) $$u_{s}=c_{0}+s\;\cdot \;u_{p}$$

where s is a parameter (the slope of the shock Hugoniot) obtained from fits to experimental data, c_0_ is the bulk speed of sound in the material and u_p_ is the particle velocity inside the compressed region behind the shock front.

By treating glycol as an incompressible gas, it is possible to use the u_s_-u_p_ state equation and the FEM-SPH method to calculate the velocity of the particles and their impact on the specimen, projectile or adjacent particles. The material constants of the glycol model for the u_s_-u_p_ equation were determined in such a way in order to obtain the convergence of the simulation and experiment results. Taking into account the above considerations, the parameters of the glycol equation of state were:

1. Speed of sound c_0_ = 343 m/s;
2. Glycol density ρ = 1250 kg/m^3^;
3. Dynamic viscosity μ = 0.002 Pa·s;
4. Compressibility parameter s = 0 (incompressible fluid was assumed).

## 3. Results and Discussion

### 3.1. Ballistic Impact Response of Samples

**Figure 8:**
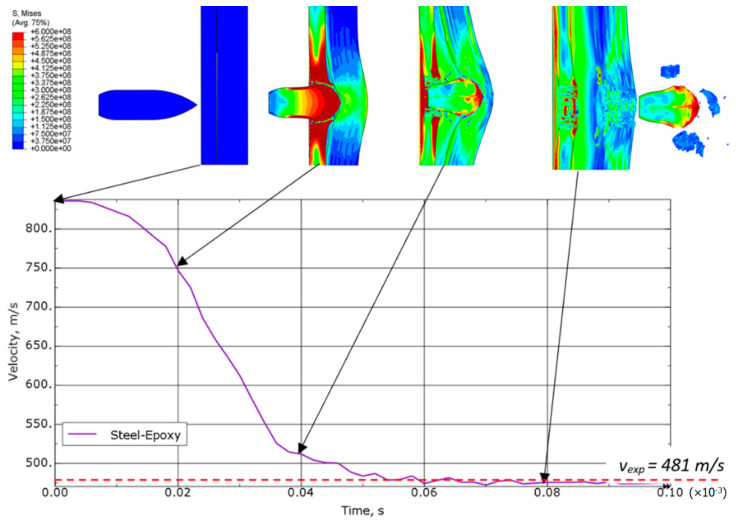
Equivalent von Mises stress distribution (in Pa) and speed drop for a steel-laminate variant.

Figure 8 shows the speed drop and the distribution of equivalent stresses during the projectile’s passage through the steel-laminate variant of sample. The impactor’s residual velocity determined experimentally is also shown as the reference. The greatest reduction in speed was observed when the projectile passed through the steel plate. The laminate plate slowed down the velocity of the projectile to a lesser extent, while at the same time the projectile’s passage through the laminate causes the oscillating character of velocity changes as a result of the destruction of aramid fiber layers. Large elastic deformations of the laminate are also visible after the projectile leaves the sample zone.

**Figure 9:**
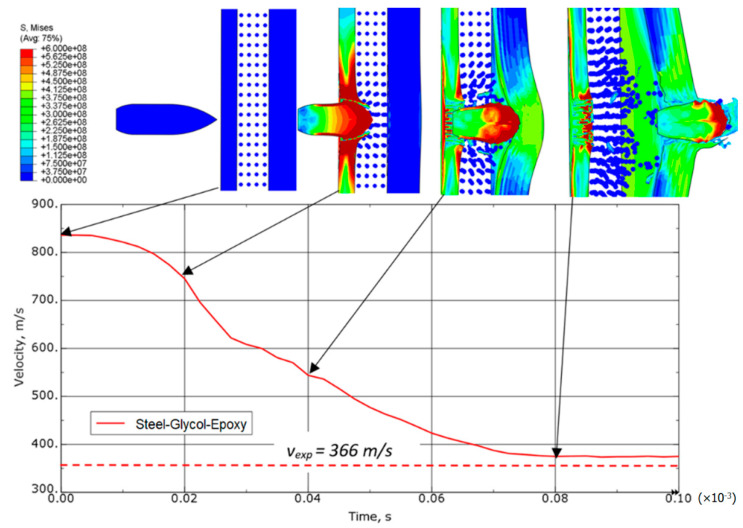
Equivalent von Mises stress distribution (in Pa) and speed drop for a steel-glycol-laminate variant.

In the variant of steel-glycol-laminate sample (Figure 9), the outlet velocity was reduced by about 20% compared to the steel-laminate variant (Figure 8). The slope of the velocity curve indicates that the projectile’s passage through the glycol layer provided more mechanical resistance than the projectile’s passage directly into the laminate layer (Figure 8). The passage of the projectile through the laminate layer is preceded by large elastic deformations.

**Figure 10:**
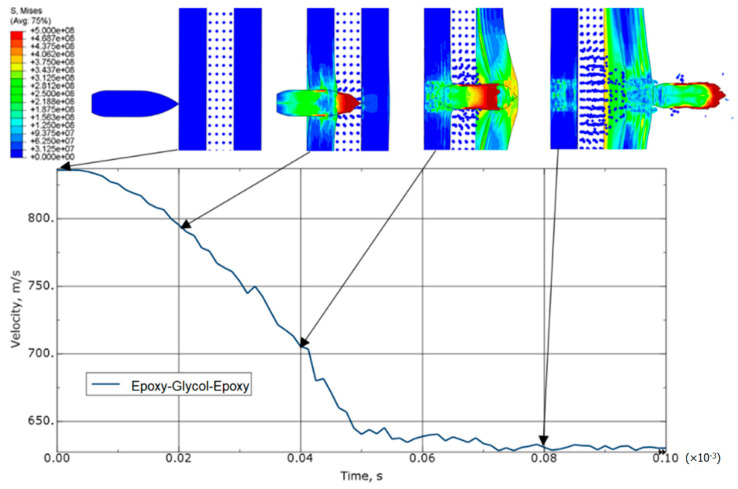
Equivalent von Mises stress distribution (in Pa) and speed drop for a laminate-glycol-laminate variant.

The passage of the projectile through the laminate-glycol-laminate sample is associated with a stable, almost uniformly sloped, velocity drop from the inlet value to the outlet value of approximately 630 m/s (Figure 10). The history of force changes showed a pulsating reaction with a series of sudden drops in load. The amplitude of the force peaks and the pulse excitation were at the same level as the projectile passed through the sample. These vibrations could be induced as a result of sudden stress shock waves in the laminate layer caused by the high velocity impact. The initial laminate layer did not represent a high mechanical resistance, therefore the projectile front was not flattened as in the previous variants (Figure 8 and Figure 9). This phenomenon made it easier for the projectile to pass through the barrier.

**Figure 11:**
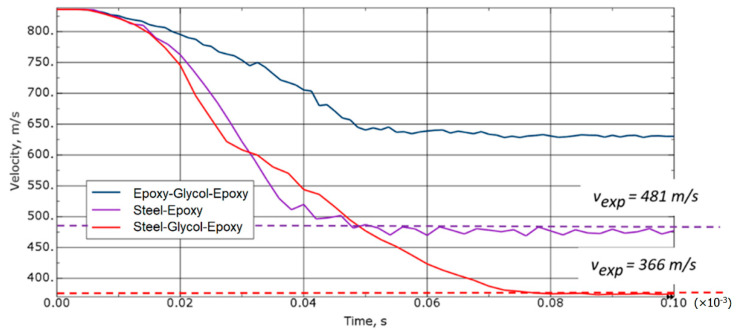
Comparison of the projectile velocity distribution depending on the variant of the sample.

Figure 11 shows a comparison of the changes in the projectile speed depending on the sample variant. In addition, the values from the experiments were marked on it in order to present the convergence of the simulations carried out. It can be seen from the chart that the greatest drop in speed occurs in the steel-glycol-laminate variant. The time taken by the projectile to completely perforate the target is termed perforation resistance. Presence of glycol-filled bag allows the projectile to be reduced by an additional 120 m/s. If two layers of laminate and glycol are used, the reduction in projectile speed is less noticeable. The simulations showed a speed decrease to the value of about 630 m/s. Referring to the steel-glycol-laminate and steel-laminate variants, it can be concluded that the laminate-glycol-laminate is the least effective.

### 3.2. Microstructural Analysis

**Figure 12:**
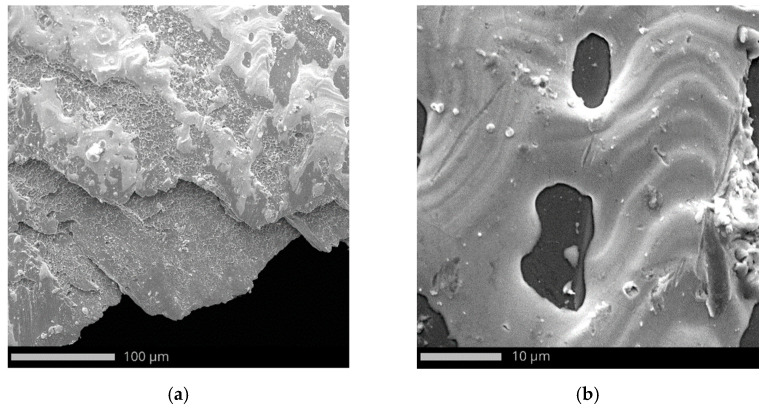
Edge of inlet with bright areas covered with lead from projectile material: (**a**) magn. ×810 and (**b**) magn. ×6300.

The microstructure of the penetration channel reveals material flow lines along with numerous shear bands (Figure 12a). The penetration of the projectile into the target causes ductile hole growth [72]. The target material was nitrogen-strengthened austenitic stainless steel. The interstitial nitrogen has the greatest solid-solution strengthening effect and stabilizes austenite [73]. Srivathsa and Ramakrishnan [74] suggested that the behavior of strain hardening determines the ballistic resistance of the steel more than the absolute value of the strength. Some of the authors stated that martensite is often observed in austenite stainless steel under quasi-static strain rate as a result of martensite transformation [75,76]. Although the content of martensite has not been tested in this work, the results of [77,78] show that martensition transformation occurs under dynamic loading which influence perforated behavior of austenitic stainless steels [79].

**Figure 13:**
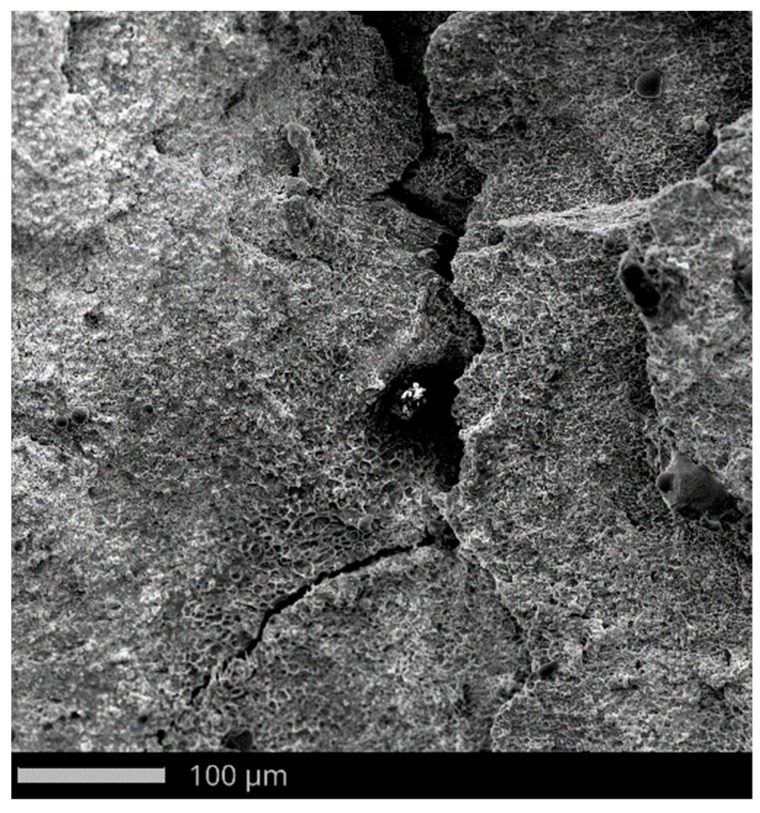
Crack at the edge of the hit.

**Figure 14:**
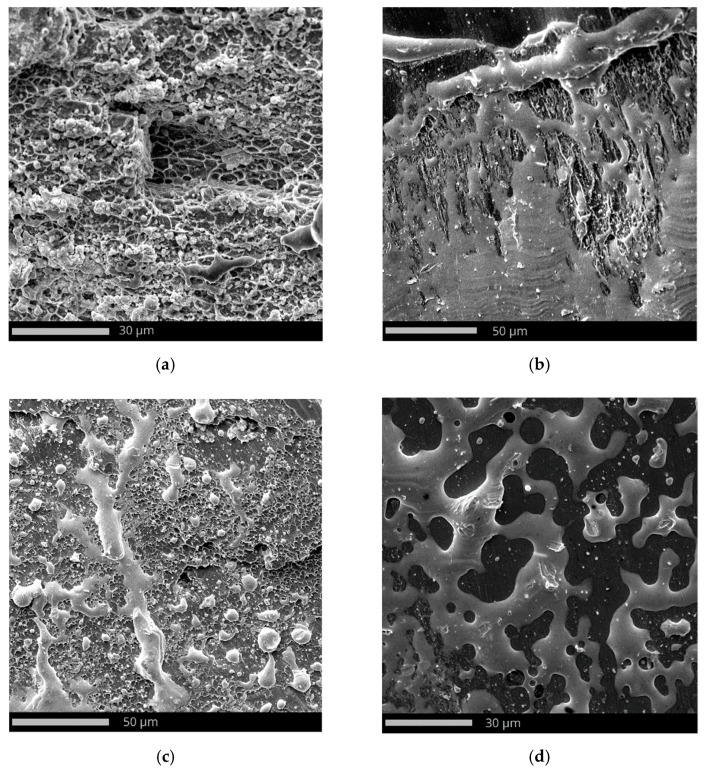
Ductile fracture of the steel plate with lead layer from projectile material at various magnifications: (**a**) ×2750, (**b**) ×1550, (**c**) ×1650, and (**d**) ×2450.

**Figure 15:**
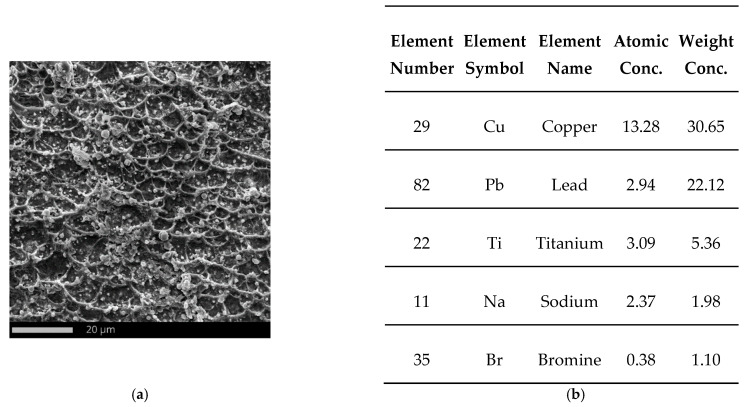
SEM micrograph of the edge of the bullet hole in the steel plate from the side of the projectile exit (**a**) and listing the most important chemical elements on the analyzed surface (**b**) determined by SEM-EDS (FOV: 86.6 µm, Mode: 15 kV—Image, Detector: SED).

**Figure 16:**
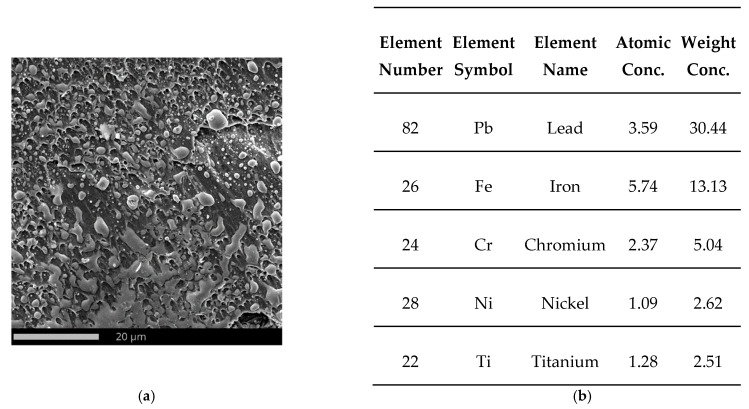
SEM micrograph of the edge of the bullet hole in the steel plate from the side of the projectile entry (**a**) and listing the most important chemical elements on the analyzed surface (**b**) determined by SEM-EDS (FOV: 86.6 µm, Mode: 15 kV—Image, Detector: SED).

The whole surface of the penetration channel is contaminated with lead from the projectile (Figure 12b). When the bullet hits the target the thin jacket is quickly eroded (thus difference in jacket calibration are almost negligible) and the soft core is able to damage the stainless steel target [80]. The high temperature generated by friction between the projectile and the target causes the lead material to heat up. In this way, a smooth surface contaminated with lead was created with the material flow lines (Figure 12b). Micro cracks are observed originating from the bottom of the crater (Figure 13). The steel plate was damaged by ductile fracture with fine dimples stretched in the direction of the projectile impact. Such dimples, clearly visible in Figure 14a, are formed as a result of the micropore growth in the material during plastic deformation [81]. This testifies to the shear nature of the material damage at the edge of the inlet of hit (Figure 14). Figure 15 and Figure 16 show the results of the EDS analysis on the edge of the bullet in the steel layer from the exit (Figure 15) and entry (Figure 16) side of the projectile. At the side of the projectile exit the hole surface is mainly contaminated with copper and lead as projectile remain. Weight concentration of the lead contaminated the the edge of the bullet hole at the side of projectile entry is similar.

## 4. Conclusions

The movement of the projectile through a fluid-filled bag creates a resistance force. 

The value of perforation resistance increases as the projectile speed decreases. Field tests have shown that the presence of glycol-filled bag increases the perforation resistance of the structure, which is also confirmed by numerical models. The FEM-SPH method made it possible to use the behavior of gases known as an equation of state to model the behavior of the fluid-filled bag. It required an appropriate approach to simplification of task analyzed. The following conclusions can be drawn from the research results:

1. For the shield variant with a glycol-filled bag between the steel and laminate plates, the inlet speed of projectile was 834 m/s on average and 366 m/s behind the sample;
2. For the variant where there was no glycol-filled bag between the steel and laminate plates, the inlet and outlet velocities were 836 m/s and 481 m/s, respectively;
3. Passage of the projectile through the laminate layer was preceded by large elastic deformations of this layer;
4. Impact of the projectile into the first steel layer leads to a large flattening of the front of the projectile and an increase in perforation resistance of subsequent layers;
5. Referring to the steel-glycol-laminate and steel-laminate variants, it can be concluded that the laminate-glycol-laminate is less effective;
6. The steel plate was damaged by ductile fracture with fine dimples stretched in the direction of the projectile impact;
7. The high temperature generated by friction between the projectile and the target causes the lead material to heat up which causes lead contamination of bullet holes.

## Figures and Tables

**Table 1:** The chemical composition (in weight %) of 1.3964 stainless steel.

C	Mn	Cr	Ni	Mo	Nb	N	Si	Fe
0.012	4.42	20.32	15.46	3.15	0.12	0.305	0.36	remainder

**Table 2:** Material properties for the core and jacket of the 7.62 mm ŁPS projectile [64].

Element of Projectile	J-C Plastic Model				J-C Failure Model		
	A	B	n	C	d_1_	d_2_	d_3_
core	234.4	413.8	0.25	0.0033	5.625	0.3	−7.2
jacket	448.2	303.4	0.15	0.0033	2.25	0.0005	−3.6

**Table 3:** Material constants in J-C material model for 1.3964 steel [63,65].

J-C Plastic Model				J-C Failure Model			
A	B	n	C	d_1_	d_2_	d_3_	u_failure_
302	1250	0.3334	0.006	0.02	0.05	0.5	0.00025

**Table 4:** Material constants for the laminate (E, Young’s modulus; ν, Poisson’s ratio; G, Kirchhoff’s modulus) [66].

E_x_	E_y_	E_z_	ν_xy_	ν_yz_	ν_xz_	G_xy_	G_yz_	G_xz_
Pa	Pa	Pa	-	-	-	Pa	Pa	Pa
1.7989 × 10^10^	1.7989 × 10^10^	1.948 × 10^9^	0.08	0.698	0.0756	1.857 × 10^9^	2.235 × 10^8^	2.235 × 10^8^

## Data Availability

The data presented in this study are available upon request from the corresponding author.

## References

[1] Christian S., Billington S. (2011). Mechanical response of PHB- and cellulose acetate natural fiber-reinforced composites for construction applications. Compos. Part B Eng..

[2] Balcıa O., Çobanb O., Borab M.Ö., Akagündüzc E., Yalçin E.B. (2017). Experimental investigation of single and repeated impacts for repaired honeycomb sandwich structures. Mater. Sci. Eng..

[3] Chai G.B., Manikandan P., Li X. (2018). A numerical study on high velocity impact behavior of titanium based fiber metal laminates. J. Compos. Sci..

[4] Pulangan M.A., Sutikno Sani M.S.M. (2019). Analysis of bulletproof vest made from fiber carbon composite and hollow glass microsphere (HGM) in absorbing energy due to projectile impact. IOP Conf. Ser. Mater. Sci. Eng..

[5] Abtew M.A., Boussu F., Bruniaux P., Loghin C., Cristian I. (2019). Ballistic impact mechanisms—A review on textiles and fibre-reinforced composites impact responses. Compos. Struct..

[6] Godzimirski J., Janiszewski J., Rośkowicz M., Surma Z. (2015). Ballistic resistance tests of multi-layer protective panels. Eksploat. I Niezawodn..

[7] D’entremont B., Grujicic M., Pandurangan B. (2012). The role of adhesive in the ballistic/structural performance of ceramic/polymer–matrix composite hybrid armor. Mater. Des..

[8] VanDerKlok A., Stamm A., Dorer J., Hu E., Auvenshine M., Pereira J.M., Xiao X. (2018). An experimental investigation into the high velocity impact responses of S2-glass/SC15 epoxy composite panels with a gas gun. Int. J. Impact Eng..

[9] Van der Werff H., Heisserer U. (2016). High-performance ballistic fibers: Ultra-high molecular weight polyethylene (UHMWPE). Advanced Fibrous Composite Materials for Ballistic Protection.

[10] Navarro C., Martinez M.A., Cortés R., Sánchez-Gálvez V. (1993). Some observations on the normal impact on ceramic faced armours backed by composite plates. Int. J. Impact Eng..

[11] Langdon G.S., Rowe L.A. The response of steel-based fibre-metal laminates to localised blast loading. Proceedings of the 17th International Conference on Composite Materials ICCM17.

[12] Langdon G.S., Nurick G.N., Cantwell W.J. (2008). The response of fibre metal laminate panels subjected to uniformly distributed blast loading. Eur. J. Mech. A/Solids.

[13] Ansari M., Chakrabarti A. (2017). Ballistic performance of unidirectional glass fiber laminated composite plate under normal and oblique impact. Procedia Eng..

[14] Yang B., He L., Gao Y. (2017). Simulation on impact response of FMLs: Effect of fiber stacking sequence, thickness, and incident angle. Sci. Eng. Compos. Mater..

[15] Sikarwar R.S., Velmurugan R., Gupta N. (2014). Influence of fiber orientation and thickness on the response of glass/epoxy composites subjected to impact loading. Compos. Part B Eng..

[16] Chen Y., Pang B., Zheng W., Peng K. (2013). Experimental investigation on normal and oblique ballistic impact behavior of fiber metal laminates. J. Reinf. Plast. Compos..

[17] Abdullah M.R., Cantwell W.J. (2006). The impact resistance of polypropylene-based fibre-metal laminates. Compos. Sci. Technol..

[18] Abdullah M.R., Cantwell W.J. (2006). The impact resistance of fiber-metal laminates based on glass fiber reinforced polypropylene. Polym. Compos..

[19] Ahmadi H., Sabouri H., Liaghat G., Bidkhori E. (2011). Experimental and numerical investigation on the high velocity impact response of GLARE with different thickness ratio. Procedia Eng..

[20] Li X., Zhang X., Guo Y., Shim V.P.W., Yang J., Chai G.B. (2018). Influence of fiber type on the impact response of titanium-based fiber-metal laminates. Int. J. Impact Eng..

[21] Sharma A.P., Khan S.H. (2018). Influence of metal layer distribution on the projectiles impact response of glass fiber reinforced aluminum laminates. Polym. Test..

[22] Sangsefidi M., Sabouri H., Mir M., Hasanpour A. (2021). High-velocity impact response of fiber metal laminates: Experimental investigation of projectile’s deformability. Thin-Walled Struct..

[23] Abdullah M.R., Cantwell W.J. (2012). The high-velocity impact response of thermoplastic-matrix fibre-metal laminates. J. Strain Anal. Eng. Des..

[24] Kikakis G.S.E., Dimou C.D., Sideridis E.P. (2017). Ballistic impact response of fiber–metal laminates and monolithic metal plates consisting of different aluminum alloys. Aerosp. Sci. Technol..

[25] Ramadhan A.A., Talib A.R.A., Rafie A.S.M., Zahari R. (2018). The Behaviour of Fibre-Metal laminates under high velocity impact loading with different stacking sequences of Al Alloy. Appl. Mech. Mater..

[26] Langdon G.S., Cantwell W.J., Nurick G.N., Jones N., Brebbin C.A. The blast performance of novel fibre-metal laminates. Structures Under Shock and Impact VIII.

[27] Balkumar K., Iver A.V., Ramasubramanian A., Devarajan K., Marimuthu P.K. (2016). Numerical Simulation of Low Velocity Impact Analysis of Fiber Metal Laminates. Mechanics Mech. Eng..

[28] Sadighi M., Alderliesten R.C., Benedictus R. (2012). Impact resistance of fiber-metal laminates: A review. Int. J. Impact Eng..

[29] Flis L. (2011). Badania odporności balistycznej pancerzy ze stali 10GHMBA na ostrzał pociskami 12.7 mm. Zesz. Nauk. Akad. Mar. Wojennej.

[30] Wang Z., Ma D., Suo T., Li Y., Manes A. (2021). Investigation into different numerical methods in predicting the response of aluminosilicate glass under quasi-static and impact loading conditions. Int. J. Mech. Sci..

[31] Johnson G.R. (2011). Numerical algorithms and material models for high-velocity impact computations. Int. J. Impact Eng..

[32] Guan Z.W., Cantwell W.J., Abdullah R. (2008). Numerical modeling of the impact response of fiber–metal laminates. Polym. Compos..

[33] Bikakis G., Tsigkros N., Sideridis E., Savaidis A. (2018). Numerical simulation of GLARE 4A fiber-metal laminates subjected to ballistic impact. MATEC Web Conf..

[34] Yaghoubi S.A., Liaw B. (2013). Effect of lay-up orientation on ballistic impact behaviors of GLARE 5 FML beams. Int. J. Impact Eng..

[35] Sitnikova E., Guan Z.W., Schleyer G.K., Cantwell W.J. (2014). Modelling of perforation failure in fibre metal laminates subjected to high impulsive blast loading. Int. J. Solids Struct..

[36] Karagiozova D., Langdon G.S., Nurick G.N., Yuen S.C.K. (2010). Simulation of the response of fibremetal laminates to localised blast loading. Int. J. Impact Eng..

[37] Zhang C., Zhu Q., Curiel-Sosa J.L., Bui T.Q. (2020). Ballistic performance and damage simulation of fiber metal laminates under high-velocity oblique impact. Int. J. Damage Mech..

[38] Ansari M., Chakrabarti A., Iqbal M. (2017). An experimental and finite element investigation of the ballistic performance of laminated GFRP composite target. Compos. Part B Eng..

[39] Ansari M.M., Chakrabarti A. (2017). Influence of projectile nose shape and incidence angle on the ballistic perforation of laminated glass fiber composite plate. Compos. Sci. Technol..

[40] Chen F., Peng Y., Chen X., Wang K., Liu Z., Chen C. (2021). Investigation of the Ballistic Performance of GFRP Laminate under 150 m/s High-Velocity Impact: Simulation and Experiment. Polymers.

[41] Vo T.P., Guan Z.W., Cantwell W.J., Schleyer G.K. (2013). Modelling of the low-impulse blast behaviour of fibre–metal laminates based on different aluminium alloys. Compos. Part B Eng..

[42] Asemani S.S., Liaghat G., Ahmadi H., Anani Y., Khodadadi A., Charandabi S.C. (2021). The experimental and numerical analysis of the ballistic performance of elastomer matrix Kevlar composites. Polym. Test..

[43] Kozłowska K., Leonowicz M. (2013). Magnetorheological fluids as a prospective component of composite armours. Compos. Theory Pract..

[44] Deshmukh S.S., McKinley G.H. (2007). Adaptive energy-absorbing material using field-responsive fluid-impregnated cellular solids. Smart Mater. Struct..

[45] Li W., Sun F., Wang P., Fan H., Fang D. (2016). A novel carbon fiber reinforced lattice truss sandwich cylinder: Fabrication and experiments. Compos. Part A.

[46] Fischer C., Braun S.A., Bourban P.E., Michaud V., Plummer C.J.G., Månson J.E. (2006). Dynamic properties of sandwich structures with integrated shear-thickening fluids. Smart Mater. Struct..

[47] Zhang Q., Qin Z., Yan R., Wei S., Zhang W., Lu S., Jia L. (2021). Processing technology and ballistic-resistant mechanism of shear thickening fluid/high-performance fiber-reinforced composites: A review. Compos. Struct..

[48] Wagner N.J., Brady J.F. (2009). Shear thickening in colloidal dispersions. Phys. Today.

[49] Chatterjee V.A., Verma S.K., Bhattacharjee D., Biswas I., Neogi S. (2019). Enhancement of energy absorption by incorporation of shear thickening fluids in 3D-mat sandwich composite panels upon ballistic impact. Compos. Struct..

[50] Xu Y. (2016). Stabbing Resistance of Soft Ballistic Body Armour Impregnated with Shear Thickening Fluid. Ph.D. Thesis.

[51] Sompoliński P. NITRONIC 50^®^, XM-19, UNS S20910—Stal Nierdzewna. https://virgamet.pl/nitronic-50-hs-shs-uns-s20910-xm-19-stal-nierdzewna-niemagnetyczna.

[52] Huebner K.H. (2001). The Finite Element Method for Engineers.

[53] Johnson G.R., Cook W.H. A Constitutive Model and Data for Metals Subjected to Large Strains, High Strain Rates. Proceedings of the 7th International Symposium on Ballistics.

[54] He A., Xie G., Zhang H., Wang X. (2013). A Comparative study on Johnson–Cook, modified Johnson–Cook and Arrhenius-Type constitutive models to predict the high temperature flow stress in 20CrMo Alloy Steel. Mater. Des..

[55] Abbasi-Bani A., Zarei-Hanzaki A., Pishbin M.H., Haghdadi N. (2014). A Comparative study on the capability of johnson–cook and arrhenius-type constitutive equations to describe the flow behavior of Mg–6Al–1Zn Alloy. Mech. Mater..

[56] Akbari Z., Mirzadeh H., Cabrera J.M. (2015). A Simple constitutive model for predicting flow stress of medium carbon microalloyed steel during hot deformation. Mater. Des..

[57] Zhan H., Wang G., Kent D., Dargusch M. (2014). Constitutive modelling of the flow behaviour of a β titanium alloy at high strain rates and elevated temperatures using the Johnson–Cook and modified Zerilli–Armstrong models. Mater. Sci. Eng..

[58] Szturomski B. (2016). Modelowanie Oddziaływania Wybuchu Podwodnego Na Kadłub Okrętu w Ujęciu Numerycznym [Modeling the Effect of the Underwater Explosion to Hull Board in a Numberic Concept].

[59] (2014). Abaqus 6.14 Theory Manual; Simulia, Dassault Systems. http://130.149.89.49:2080/v6.14/pdf_books/ANALYSIS_3.pdf.

[60] Kohnke P. (1999). Ansys Theory Reference, Release 5.6. http://research.me.udel.edu/~lwang/teaching/MEx81/ansys56manual.pdf.

[61] Banerjee A., Dhar S., Acharyya S., Datta D., Nayak N. (2015). Determination of Johnson Cook material and failure model constants and numerical modelling of charpy impact test of armour steel. Mater. Sci. Eng. A.

[62] Garbarz B., Marcisz J., Burian W., Wiśniewski A. (2011). Mechanizmy odkształcenia dynamicznego w ultrawytrzymałych stalach nanostrukturalnych przeznaczonych na pancerze. Probl. Tech. Uzbroj..

[63] Kiciński R. (2020). Analiza i Modelowanie Odporności na Przebicie Okrętowych Konstrukcji Osłonowych, (Strength analysis of a Ships Ballistic Covers). Rozprawa Doktorska. Ph.D. Thesis.

[64] Carbajal L., Jovicic J., Kuhlmann H. (2011). Assault riffle bullet-experimental characterization and computer (FE) Modeling. Experimental and Applied Mechanics.

[65] Jurczak W., Szturomski B., Świątek K. (2018). Determination of dynamic characteristics of austenitic steel to be utilized in fem simulation and its verification. Sci. J. Pol. Nav. Acad..

[66] Hiermaier S.J., Riedel W., Clegg R.A., Hayhurst C.J. (1999). Advanced Material Model for Hypervelocity Impact Simulations.

[67] Lucy L.B. (1997). A Numerical approach to the testing of the fission hypothesis. Astron. J..

[68] Monaghan J.J., Gingold R.A. (1997). Smoothed particle hydrodynamics: Theory and applications to non-spherical stars. R. Astron. Soc..

[69] Danielewicz A. (2016). Metoda SPH+MES Na Przykładzie Symulacji Wzmocnienia Podłoża Gruntowego Metodą Wymiany Dynamicznej.

[70] Bohdal L., Kukielka L., Świłło S., Radchenko A.M., Kułakowska A. (2019). Modelling and Experimental Analysis of Shear-Slitting Process of Light Metal Alloys Using FEM, SPH and Vision-Based Methods.

[71] Kerley G.I. (2006). The Linear US-UP Relation in Shock-Wave Physics.

[72] Sundaram S.K., Bharath A., Aravind B. (2022). Influence of target dynamics and number of impacts on ballistic performance of 6061-T6 and 7075-T6 aluminum alloy targets. Mech. Based Des. Struct. Mach..

[73] Pickering F.B. (1976). Physical metallurgy of stainless steel developments. Int. Met. Rev..

[74] Srivathsa B., Ramakrishnan N. (1999). Ballistic performance maps for thick metallic armour. J. Mater. Process. Technol..

[75] Byun T.S., Hashimoto N., Farrell K. (2004). Temperature dependence of strain hardening and plastic instability behaviors in austenitic stainless steels. Acta Mater..

[76] Hamada A.S., Karjalainen L.P., Misra R.D.K., Talonen J. (2013). Contribution of deformation mechanisms to strength and ductility in two Cr–Mn grade austenitic stainless steels. Mater. Sci. Eng. A.

[77] Rusinek A., Rodriguez-Martinez J.A., Pesci R., Capelle J. (2010). Experimental characterisation and modelling of the thermo-viscoplastic behaviour of steel AISI 304 within wide ranges of strain rate at room temperature. J. Theor. Appl. Mech..

[78] Zaera R., Rodríguez-Martínez J.A., Casado A., Fernández-Sáez J., Rusinek A., Pesci R. (2012). A constitutive model for analyzing martensite formation in austenitic steels deforming at high strain rates. Int. J. Plast..

[79] Jia B., Rusinek A., Bahi S., Berner R., Pesci R., Bendarma A. (2019). Perforation Behavior of 304 Stainless Steel Plates at Various Temperatures. J. Dyn. Beh. Mater..

[80] Giglio M., Gilioli A., Manes A., Peroni L., Scapin M. (2012). Investigation about the influence of the mechanical properties of lead core and brass jacket of a NATO 7.62 mm ball bullet in numerical simulations of ballistic impacts. EPJ Web Conf..

[81] Wciślik W., Pała R. (2021). Some microstructural aspects of ductile fracture of metals. Materials.

